# Treatment with a *Lactococcus lactis* that chromosomally express *E. coli cfaI* mitigates salivary flow loss in a Sjögren’s syndrome-like disease

**DOI:** 10.1038/s41598-023-46557-3

**Published:** 2023-11-09

**Authors:** Ali Akgul, Christian Furlan Freguia, Massimo Maddaloni, Carol Hoffman, Alexandria Voigt, Cuong Q. Nguyen, Neil A. Fanger, Gary R. Fanger, David W. Pascual

**Affiliations:** 1 Department of Infectious Diseases and Immunology, University of Florida, Gainesville, FL USA; 2 Rise Therapeutics, Rockville, MD USA; 3 Virtici, Seattle, WA USA

**Keywords:** Immunology, Diseases

## Abstract

Sjögren’s Syndrome (SjS) results in loss of salivary and lacrimal gland excretion due to an autoimmune attack on these secretory glands. Conventional SjS treatments address the symptoms, but not the cause of disease. Recognizing this deficit of treatments to reverse SjS disease, studies were pursued using the fimbriae from enterotoxigenic *E. coli*, colonization factor antigen I (CFA/I), which has anti-inflammatory properties. To determine if CFA/I fimbriae could attenuate SjS-like disease in C57BL/6.NOD-*Aec1Aec2* (SjS) females, the *Lactococcus lactis* (LL) 301 strain was developed to chromosomally express the *cfaI* operon. Western blot analysis confirmed CFA/I protein expression, and this was tested in SjS females at different stages of disease. Repeated dosing with LL 301 proved effective in mitigating salivary flow loss and in reducing anti-nuclear antibodies (ANA) and inflammation in the submandibular glands (SMGs) in SjS females and in restoring salivary flow in diseased mice. LL 301 treatment reduced proinflammatory cytokine production with concomitant increases in TGF-β^+^ CD25^+^ CD4^+^ T cells. Moreover, LL 301 treatment reduced draining lymph and SMG follicular T helper (Tfh) cell levels and proinflammatory cytokines, IFN-γ, IL-6, IL-17, and IL-21. Such evidence points to the therapeutic capacity of CFA/I protein to suppress SjS disease and to have restorative properties in combating autoimmune disease.

## Introduction

Sjögren’s Syndrome (SjS) is a chronic, progressive autoimmune disease characterized by inflammatory cell infiltration of the salivary and lacrimal glands, resulting in acinar epithelial cell atrophy, cell death, and loss of exocrine function (reviewed in^1–3^). The disease incidence is estimated to be 0.1–3% of the general population^4–6^ occurring primarily in postmenopausal women^4,5,7^. SjS is a debilitating disease affecting as many as 3.1 million individuals in the US^1–3,5,6^ with women being nine times more likely to be afflicted than men^2,4,5,7^. The etiology of SjS remains unclear. The C57BL/6.NOD-*Aec1Aec2* mouse model was derived based on the observation of SjS-like disease in diabetic NOD mice^8–11^. The responsible genes associated with SjS, but free from those responsible for diabetes, were inserted into B6 mice to recapitulate SjS^10^. This model has the advantage of exhibiting many of the salivary gland pathology observed with human SjS patients, and increased production of anti-nuclear antibodies (ANAs). Hence, studies with various mouse models, including the C57BL/6.NOD-*Aec1Aec2* mouse, are beginning to define both the innate and adaptive immune responses during development and onset of SjS-like disease^8–10^.

The development of therapeutics for SjS poses significant challenges, in part because of the heterogeneity of clinical disease. Few studies have addressed the critical issue of identifying interventions to stop or reverse the destructive autoimmune process. Currently, no therapeutics address the cause of SjS, but instead treatments consist of replacement therapies such as artificial saliva and eye lubricants or immunosuppressive agents^12,13^. B cell-directed therapies, e.g., Rituximab (anti-CD20 mAb), have yielded promising, but conflicting results^14–18^, and are unable to alleviate patients’ dryness and fatigue^19,20^. CTLA-4 protein (Abatacept) showed some reduction in gland pathology^21^, and enhancement of salivary flow in a limited number of SjS patients^22^. Abatacept was recently shown to reduce serum IL-21 and follicular T helper (Tfh) cells in SjS patients^23,24^, and to reduce SjS symptoms^25,26^.

Originally conceived as a diarrheal vaccine for humans^27–30^, colonization factor antigen I (CFA/I) from enterotoxigenic *E. coli* (ETEC) was found by our lab to be potently effective in suppressing experimental models for multiple sclerosis^31–33^, arthritis^34–37^, and type 1 diabetes^38,39^. In fact, purified CFA/I fimbriae, given orally or nasally, can effectively attenuate inflammation^36^. The *cfa1* operon has been successfully engineered into a *Lactococcus lactis* strain, and referred to as *L. lactis*-CFA/I (LL-CFA/I). By retaining its inhibitory activity, oral administration of LL-CFA/I protects against arthritis^37^. Of note, our results show that LL-CFA/I can attenuate genetically induced SjS via the stimulation/reactivation of diverse regulatory T cells (Tregs) producing TGF-β and IL-10^40^. Moreover, LL-CFA/I was found to be therapeutic for ongoing SjS.

The use of *L. lactis* for delivery of biologics and CFA/I protein has several advantages: (1) oral dosing of targeted biological immunotherapy which traditionally require infusion or frequent injections; (2) controllable dosing as *L. lactis* does not colonize the human gastrointestinal tract^41,42^, and our own studies in mice confirm its transient presence in tissues for < 48 h; and (3) pharmacodynamically optimized delivery of constitutively expressed CFA/I protein for engagement of microbiome-associated immune regulatory pathway. The fact that *L. lactis* does not colonize the gut^41,43^ is ideal for an immunotherapeutic as it enables a ‘tunable’ therapeutic strategy wherein identification of key mechanism-driving biomarkers, as discussed herein in the context of CFA/I protein, become critical for effective and efficient clinical development. Importantly, *L. lactis*, in the context of synthetic biology approaches to deliver CFA/I, allows for utilization of precision targeting methods, not capable using natural commensal strain strategies.

To advance clinical translation, an *L. lactis* line was generated to chromosomally express *cfaI* (referred to as LL 301), and LL 301 showed equivalent efficacy to LL-CFA/I, the precursor *L. lactis* line that expresses CFA/I from an episomal plasmid. Chromosomal insertion of the *cfa/I* operon increases stability of the line, as propagation no longer requires antibiotic selection, and relieves concerns regarding episomal transfer in vivo. When testing LL 301 in the genetic model for SjS using C57BL/6.NOD-*Aec1Aec2* mice, our results show that prophylactic treatments proved effective in salivary flow mitigation, and therapeutic interventions restored salivary flow. Such changes from the diseased state were accomplished via the stimulation of Tregs and suppression of effector T (Teff) cells including Tfh cells.

## Results

### Oral intervention with LL 301 retains salivary flow in SjS mice

**Figure 1:**
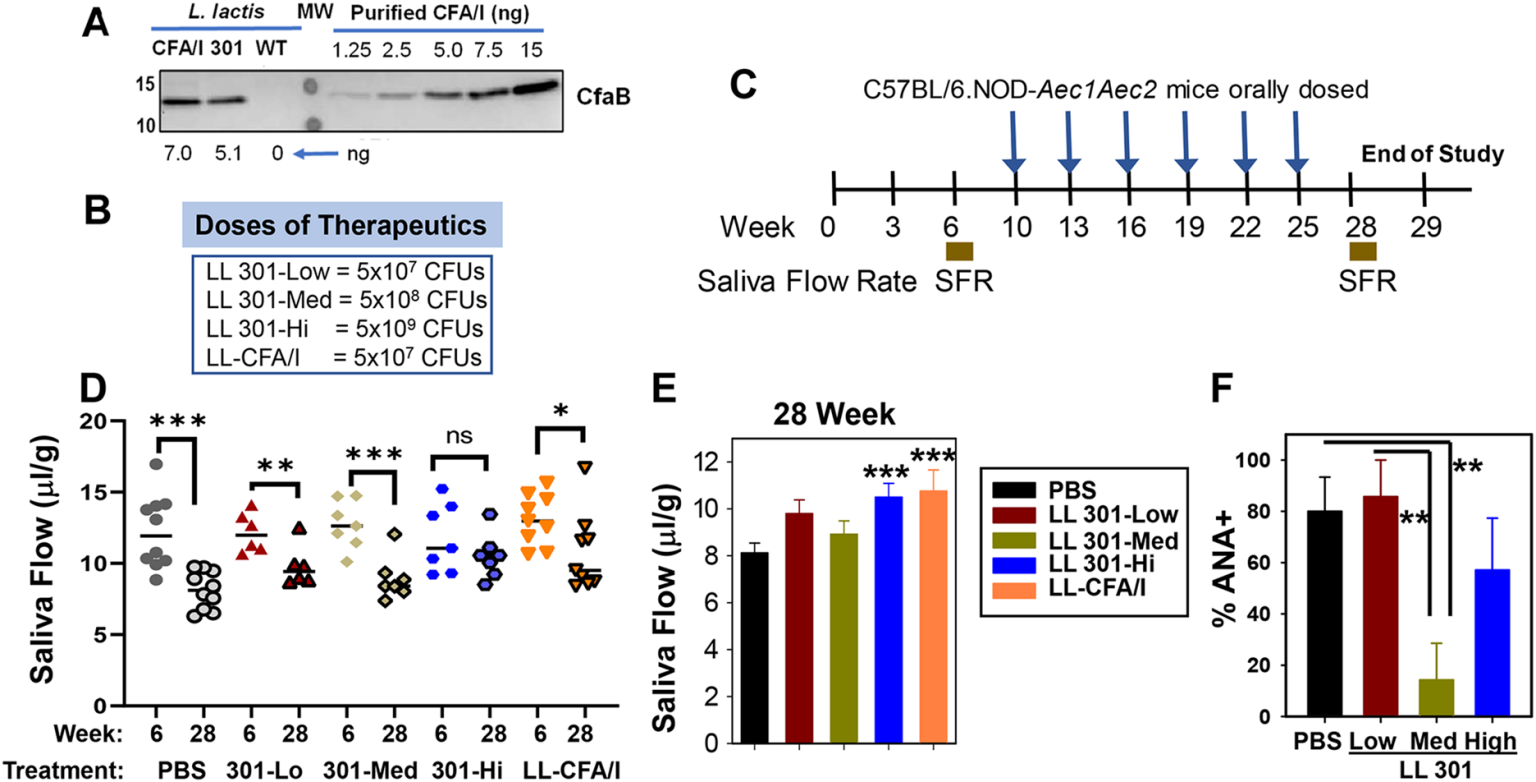
Oral treatments with *Lactococcus lactis* strain 301 (LL 301) protect against the development of SjS. (**A**) LL 301 with the *E. coli cfaI* operon incorporated into its chromosome produces CFA/I protein similar to that generated by episomally produced by the LL-CFA/I strain. A Western blot was performed on whole bacterial extracts from LL 301 electrophoresed in SDS polyacrylamide gel, and compared to bacterial extracts from LL-CFA/I and wild-type (WT) LL. The CfaB subunit was detected with a rabbit anti-CFA/I fimbriae (produced in-house) and migrated with similar molecular weight (MW) as purified CFA/I fimbriae. The amount produced by 10^9^ bacteria is estimated from densiometric scan of purified CFA/I fimbriae. The original Western blot is presented in Supplemental Fig. 1. (**B**) The doses and (**C**) treatment regimen of C57BL/6.NOD-*Aec1Aec2* (SjS) mice used in this study is provided. Individual salivary flow rate (SFR) were measured prior to treatment at 10 weeks of age and after completion of treatments at 25 weeks of age. Groups of 10-week-old SjS (8–9 mice/group) females were orally dosed with 5 × 10^7^ (low dose) 5 × 10^8^ (medium dose), or 5 × 10^9^ CFUs (high dose) of LL 301, 5 × 10^7^ LL-CFA/I, or phosphate-buffered saline (PBS). Additional doses were administered to the mice every 3 weeks. SFR measurements (**D**) relative to 6 weeks of age and (**E**) relative to PBS-treated mice are shown; ***P < 0.001, **P < 0.01, *P < 0.05 versus 6 week or PBS-treated groups; ns = not significant. (**F**) The percentage of animals being serum anti-nuclear antibody (ANA) positive was determined for PBS- and LL 301-treated mice; **P < 0.02 versus the indicated group.

LL 301 was generated to have a *Lactococcus lactis* strain expressing CFA/I protein without reliance upon episomal expression as in *L. lactis*-CFA/I (LL-CFA/I). A Δ*thyA L. lactis* was produced by double homologous recombination via insertion of the *cfaI* operon replacing the thymidylate synthase (*thyA*) gene. Production levels of the major subunit, CfaB, by LL 301 was confirmed as evidenced by Western blot analysis (Fig. 1A).

In evaluating LL 301’s efficacy relevant to LL-CFA/I to mitigate salivary flow loss in C57BL/6.NOD-*Aec1Aec2* (SjS) mice, baseline individual saliva flow rate (SFR) for all groups of 6 week (wk)-old females were obtained. Treatments of SjS mice were initiated at 10 weeks of age dosing at 3-week intervals for a total of six doses with PBS or one of three doses of LL 301: 5 × 10^7^ (low), 5 × 10^8^ (medium), or 5 × 10^9^ (high) CFUs (Fig. 1B, C). As a positive control, one group of mice was given 5 × 10^7^ CFUs of LL-CFA/I. SFR measurements were conducted at 28 weeks of age, three weeks after the last dose (Fig. 1D, E). Both the high dose LL 301 and LL-CFA/I proved significantly effective in retaining salivary flow relative to PBS-dosed mice (Fig. 1E). Treatments with the medium dose of LL 301 also proved effective in reducing the frequency of mice exhibiting ANA titers (Fig. 1F).

### LL 301 treatment reduces proinflammatory cytokine production with a concomitant increase in anti-inflammatory cytokines

**Figure 2:**
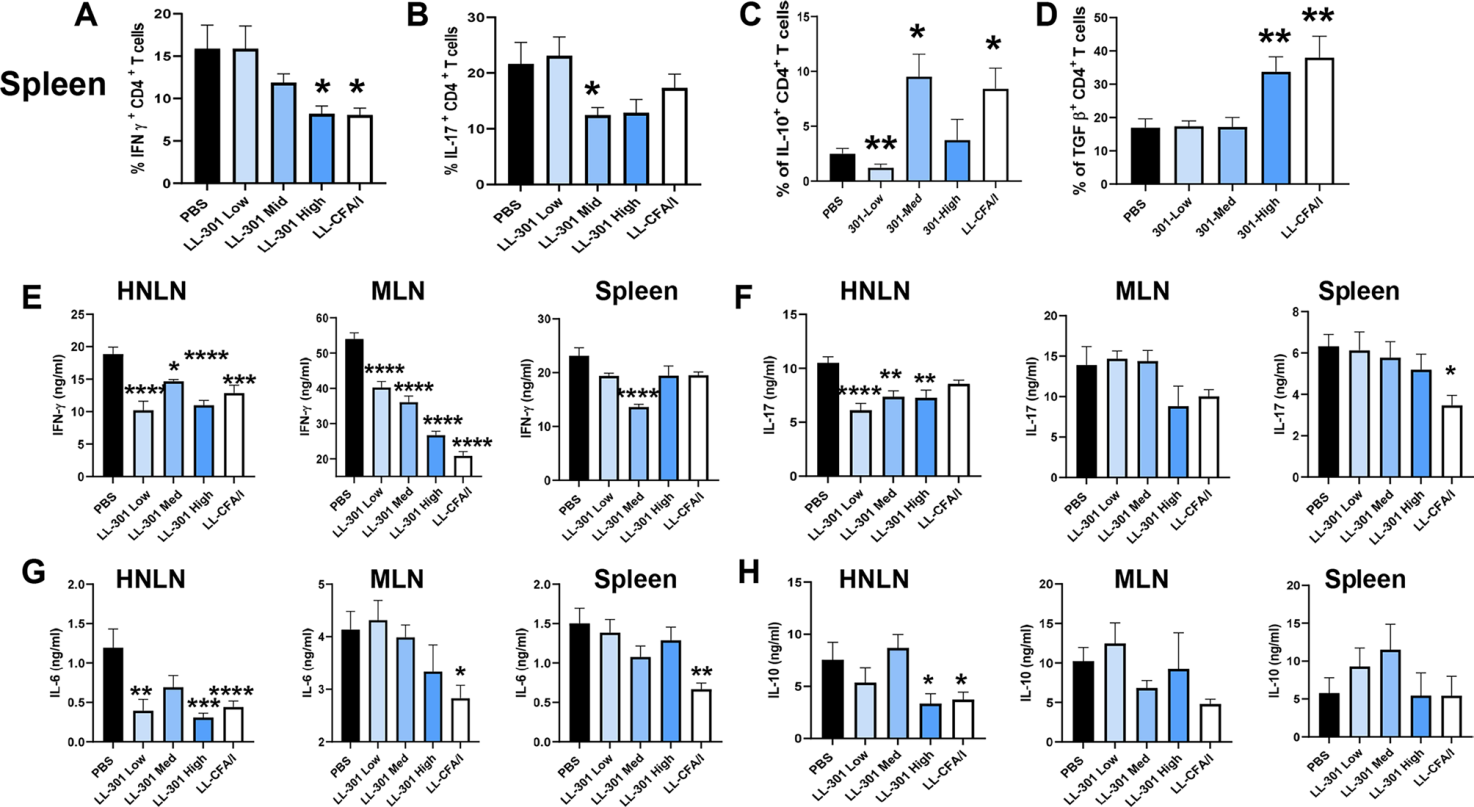
Oral treatments with LL 301 reduce inflammatory cells and augments anti-inflammatory CD4^+^ T cells. The percentages of splenic (**A**) IFN-γ^+^, (**B**) IL-17^+^, (**C)** IL-10^+^, and (**D**) TGF-β^+^ CD4^+^ T cells are depicted from the same mice in Fig. 1. (**E–H**) Purified lymphocytes from the head and neck lymph nodes (HNLNs), mesenteric LNs (MLNs), and spleens from each treatment group were stimulated with anti-CD3 and anti-CD28 mAbs for 4 days for cytokine ELISA. Individual culture supernatants were analyzed by cytokine-specific ELISAs for production of (**E**) IFN-γ, (**F**) IL-17, (**G**) IL-6, and (**H**) IL-10. Depicted are the means ± SEM; ****P < 0.001, ***P < 0.001, **P < 0.01, *P < 0.05 versus PBS-dosed mice.

The mitigation of SFR loss would be expected to result from inflammatory cell arrest. The study was terminated at week 29, and the levels of IFN-γ- and IL-17-producing CD4^+^ T cells in the spleen were measured. The high dose LL 301 and LL-CFA/I effectively reduced the percentage of IFN-γ^+^ CD4^+^ T cells (Fig. 2A). Changes in IL-17^+^ CD4^+^ T cells were observed only for the LL 301 medium dose (Fig. 2B); however, increases in IL-10^+^ CD4^+^ T cells were obtained with the LL 301 medium dose and with LL-CFA/I (Fig. 2C). The percentage of TGF-β^+^ CD4^+^ T cells also increased in SjS mice treated with LL 301 high dose and LL-CFA/I (Fig. 2D).

Additional cytokine analysis was performed on anti-CD3 plus anti-CD28 mAb treatments to measure if head and neck lymph nodes (HNLNs), mesenteric LNs (MLNs) and splenic T cells exhibit anti-inflammatory cytokine capacity. All of the HNLN and MLN treatment groups showed significant reduction in IFN-γ production compared to PBS-dosed SjS mice (Fig. 2E). The LL 301 medium dose treatment group showed significant reduction of IFN-γ production (Fig. 2E). IL-17 was significantly reduced by all LL 301 treatment doses by HNLN lymphocytes and by splenic lymphocytes from the LL-CFA/I-treated mice (Fig. 2F). IL-6 production was also significantly reduced by HNLN lymphocytes from SjS mice treated with the LL 301 low and high doses, and LL-CFA/I-treated mice showed significant reduction in IL-6 from the HNLN, MLN, and splenic lymphocytes (Fig. 2G). IL-10 production remained relatively unchanged among the three lymphoid tissues except for the HNLN lymphocytes from the LL 301 high dose and LL-CFA/I groups showed a significant reduction (Fig. 2H).

### Salivary flow is mitigated by LL 301 treatments and not with WT LL

**Figure 3:**
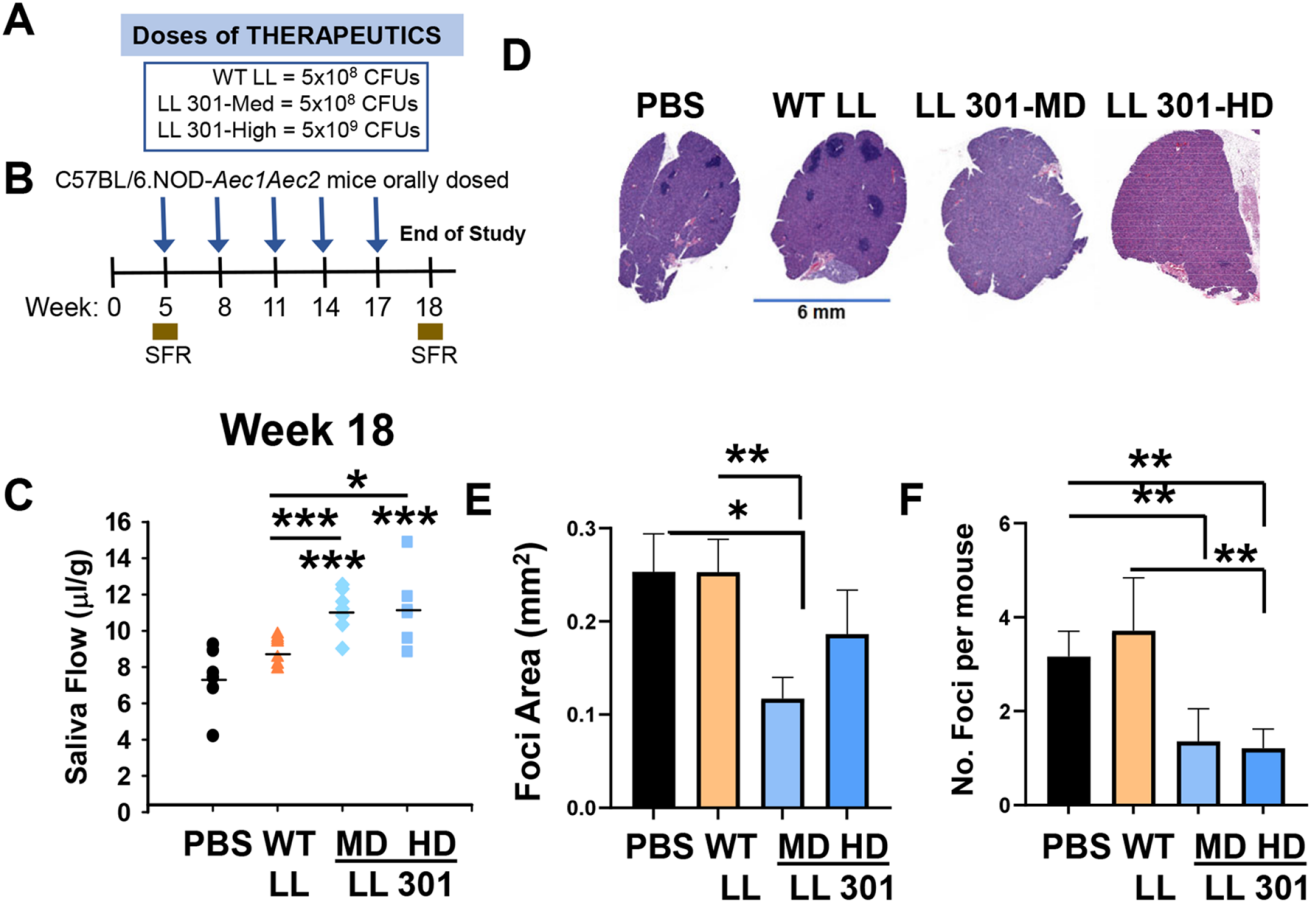
Oral treatments with LL 301 protect against the development of SjS retaining SFR and reducing gland inflammation. The (**A**) doses and (**B**) treatment regimen used to treat SjS mice in this study are provided. Individual baseline SFR was obtained prior to treatment onset and upon termination of the study at 18 weeks of age. Groups of 5-week-old SjS (6–8 mice/group) females were orally dosed with 5 × 10^8^ (medium dose; MD) or 5 × 10^9^ CFUs (high dose; HD) of LL 301, 5 × 10^8^ CFUs of wild-type (WT) LL, or PBS. Additional doses were administered to the mice every 3 weeks for a total of 5 doses. (**C**) SFR measurements relative to PBS-treated mice are shown; ***P < 0.001, *P < 0.05, one-way ANOVA followed by Dunnett’s multiple comparisons test was performed. (**D**) At 19 weeks of age, submandibular glands (SMGs) were formalin fixed and stained with hematoxylin and eosin to determine extent of inflammatory cell infiltration. Representative images of stained tissues at × 20 magnification. Infiltrated regions were drawn for area determinations and calculated by using the Aperio ImageScope software. Focus score of infiltrates were determined by using (**E**) average focus size in area and (**F**) the number of foci in 4 mm^2^; **P < 0.01, *P < 0.05 versus PBS- or WT LL-treated mice are shown. One-way ANOVA followed by Dunnett’s multiple comparisons test was performed.

Given the positive outcome of the previous findings using LL 301, the study was repeated using a medium (5 × 10^8^ CFUs) and high (5 × 10^9^ CFUs) doses of LL 301 compared to groups of SjS females treated with WT LL (5 × 10^8^ CFUs) and PBS vehicle (Fig. 3A). Treatments were initiated at 5 weeks of age and additional doses administered at 3 week intervals until 17 weeks of age (Fig. 3B). SFRs were measured for individual mice, and showed that both doses of LL 301 were effective mitigating salivary flow in contrast to WT LL or PBS-vehicle treated SjS females (Fig. 3C). No significant differences in SFR were observed between PBS- and WT LL-treated SjS females. H&E staining was performed on paraffin-embedded submandibular glands (SMGs) from individual mice of each treatment group (Fig. 3D), and foci area and number of foci were quantified (Fig. 3E, F). The medium LL 301 dose showed ~ 50% reduction in foci area compared to vehicle- or WT LL-treated SjS mice (Fig. 3E). Both doses of LL 301 proved effective in reducing the number of foci in the SMGs, and the LL 301 high dose also significantly lessened the number of SMG foci relative to WT LL-treated mice (Fig. 3F).

**Figure 4:**
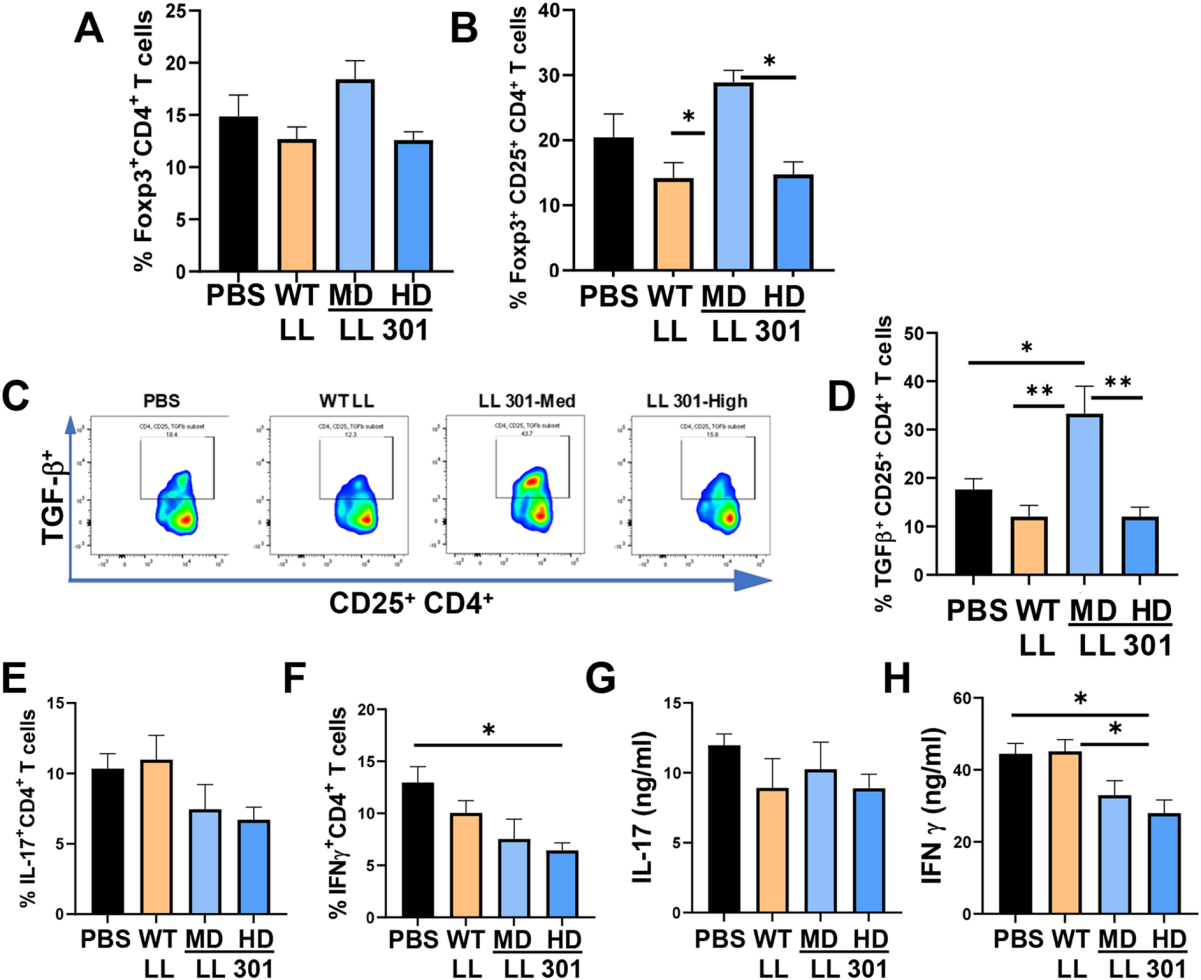
LL 301 treatments enhances TGF-β^+^ regulatory T cells (Tregs). The same mice from Fig. 3 were analyzed for Tregs in the spleen by flow cytometry. One week after SFR measurements, splenic lymphocytes from individual mice from each treatment group were measured for percentage of (**A**) Foxp3^+^ CD4^+^ and (**B**) Foxp3^+^ CD25^+^ CD4^+^ T cells. (**C**) The CD25^+^ CD4^+^ T cells were further analyzed for TGF-β expression and (**D**) percentages. LL 301 treatment (**E**) did not significantly reduce the percentage of IL-17^+^ CD4^+^ T cells, but (**F**) did reduce the percentage of IFN-γ^+^ CD4^+^ T cells; **P < 0.01, *P < 0.05 relative to the indicated treatment group. (**G,H**) Treatment with LL 301 suppresses IFN-γ production. Splenic lymphocytes from each treatment group were stimulated with anti-CD3 and anti-CD28 mAbs for 4 days, and culture supernatants measured for the production of (**G**) IL-17 and (**H**) IFN-γ by cytokine-specific ELISAs. Depicted are the means ± SEM; *P < 0.05 versus PBS-dosed mice or indicated treatment group.

Additional analysis was conducted to determine if LL 301 was capable of eliciting regulatory T cells (Tregs). No significant differences in splenic Foxp3^+^ CD4^+^ T cells or Foxp3^+^ CD25^+^ CD4^+^ T cells were observed relative to vehicle-treated mice (Fig. 4A, B); however, the LL 301 medium dose effectively enhanced Foxp3^+^ CD25^+^ CD4^+^ T cells relative to WT LL-treated SjS females (Fig. 4B). The CD25^+^ CD4^+^ T cells were further analyzed for expression of TGF-β, and the LL 301 medium dose showed significantly increased percentages of splenic TGF-β^+^ CD25^+^ CD4^+^ T cells relative to PBS- or WT LL-treated SjS females (Fig. 4C, D). Neither dose of LL 301 impacted the frequency of IL-17^+^ CD4^+^ T cells (Fig. 4E) or IL-17 production from restimulated splenic lymphocytes (Fig. 4G). In contrast, the high dose of LL 301 reduced the percentage of splenic IFN-γ^+^ CD4^+^ T cells (Fig. 4F) and reduced IFN-γ production from restimulated splenic lymphocytes compared to vehicle- or WT LL-treated mice (Fig. 4H).

### Oral treatment with LL 301 is therapeutic in restoring salivary flow, and reducing inflammatory cytokine production.

**Figure 5:**
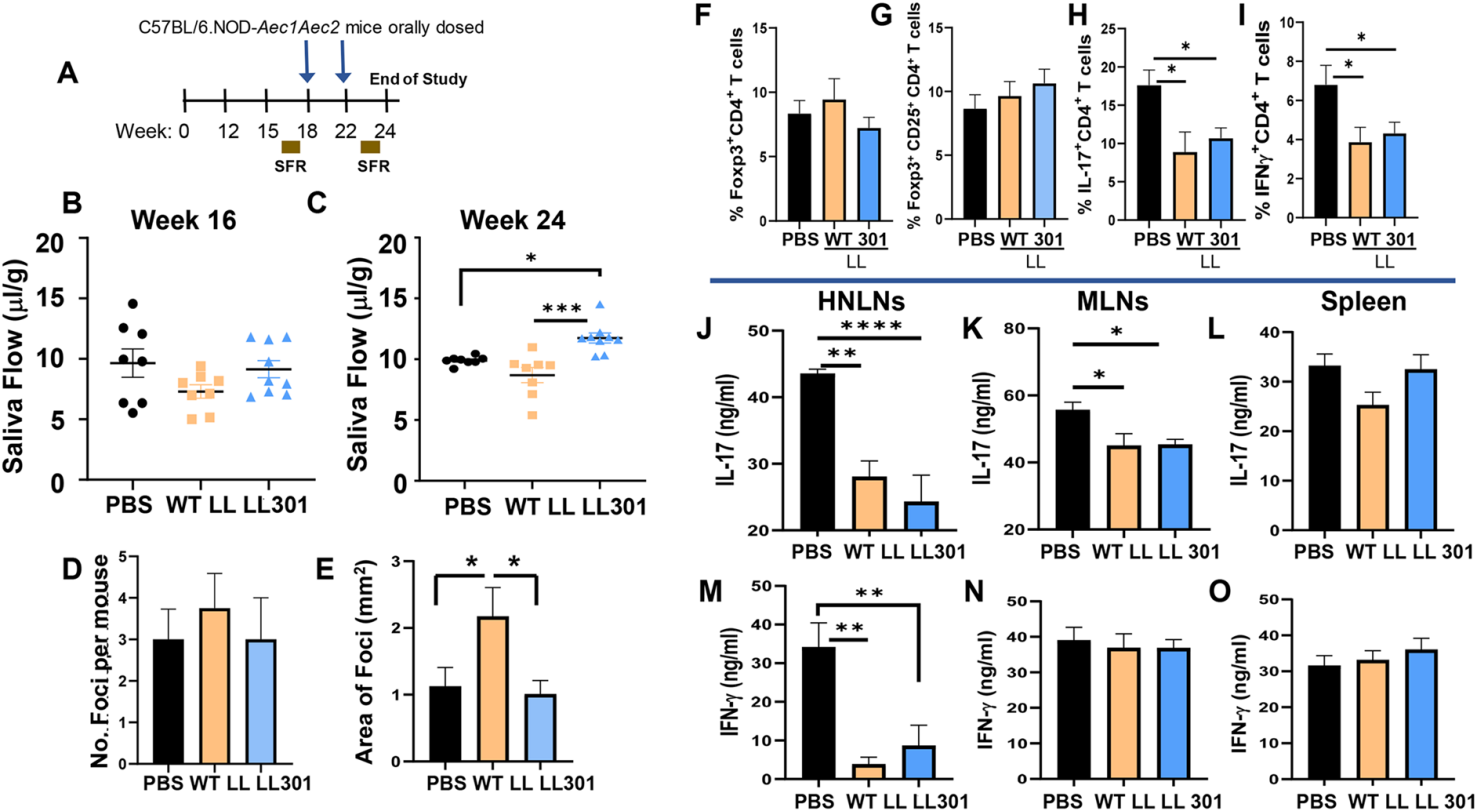
Oral treatments with LL 301 restore salivary flow in diseased SjS mice. (**A**) Groups of 16 week-old SjS females (8/group) were orally treated with PBS, 5 × 10^9^ CFUs WT LL, or 5 × 10^9^ CFUs LL 301, and a second dose administered 4 weeks later. SFR measurements were taken (**B**) prior and (**C**) after second treatment; ***P < 0.001, *P < 0.05 versus the indicated group. Study was terminated 24 weeks of age, and SMGs were formalin fixed and stained with hematoxylin and eosin to determine extent of inflammatory cell infiltration. Images of stained tissues at × 20 magnification were examined for infiltrated regions using the Aperio ImageScope software as described in Fig. 3. Focus score of infiltrates were determined by using (**D**) the number of foci and (**E**) focus size in area; *P < 0.05 versus WT LL-treated mice are shown. One-way ANOVA followed by Dunnett’s multiple comparisons test was performed. Splenic lymphocytes were analyzed for percentages of (**F**) Foxp3^+^ CD4^+^ and (**G**) Foxp3^+^ CD25^+^ CD4^+^ T cells, and for the percentages of (**H**) IL-17^+^ and (**I**) IFN-γ^+^ CD4^+^ T cells; *P < 0.05 relative to PBS- or WT LL-dosed mice are shown. (**J,M**) Purified HNLN, (**K,N**) MLN, and (**L,O**) splenic lymphocytes from PBS-, WT LL-, and LL 301-treated mice were stimulated with anti-CD3 and anti-CD28 mAbs for 4 days. Culture supernatants were analyzed for production of (**J–L**) IL-17 and (**M–O**) IFN-γ by cytokine-specific ELISAs. Depicted are the means ± SEM; ****P < 0.0001, **P < 0.01, *P < 0.05 versus the indicated group.

To discern LL 301’s therapeutic potential (Fig. 5A), groups of SjS females were measured for their SFRs at 16 weeks of age, and then the mice were equally distributed among the three treatment groups (Fig. 5B). At 18 and 22 weeks of age, mice were treated with PBS, 3 × 10^9^ CFUs WT LL, or 3 × 10^9^ CFUs LL 301, and SFRs measured again at 24 weeks of age (Fig. 5A). After just two doses of LL 301, SjS females showed a significant increase in their SFRs, but not those treated with PBS nor WT LL (Fig. 5C). Although there were no significant differences in the number of foci in their SMGs relative to PBS-treated mice (Fig. 5D), the foci area was significantly increased in WT LL-treated mice (Fig. 5E). Upon termination of the mice at 24 weeks of age, splenic lymphocytes were examined for the presence of Tregs, but no differences in Foxp3^+^ CD4^+^ T cells nor Foxp3^+^ CD25^+^ CD4^+^ T cells were found (Fig. 5F, G). However, treatment with LL 301 and WT LL did show significant reductions in the percentage of IL-17^+^ CD4^+^ and IFN-γ^+^ CD4^+^ T cells (Fig. 5H, I). Total lymphocytes from the HNLNs, MLNs, and spleen were restimulated, and collected supernatants measured for IL-17 and IFN-γ production. Significant reductions in HNLN and MLN IL-17 were observed by LL 301- and WT LL-treated groups (Fig. 5J, K). HNLN IFN-γ production was also significantly reduced by the LL 301- and WT LL-treated groups (Fig. 5M). Splenic IL-17 and IFN-γ levels remained unchanged.

### Treatment with LL 301 suppresses Tfh cells in SjS mice

**Figure 6:**
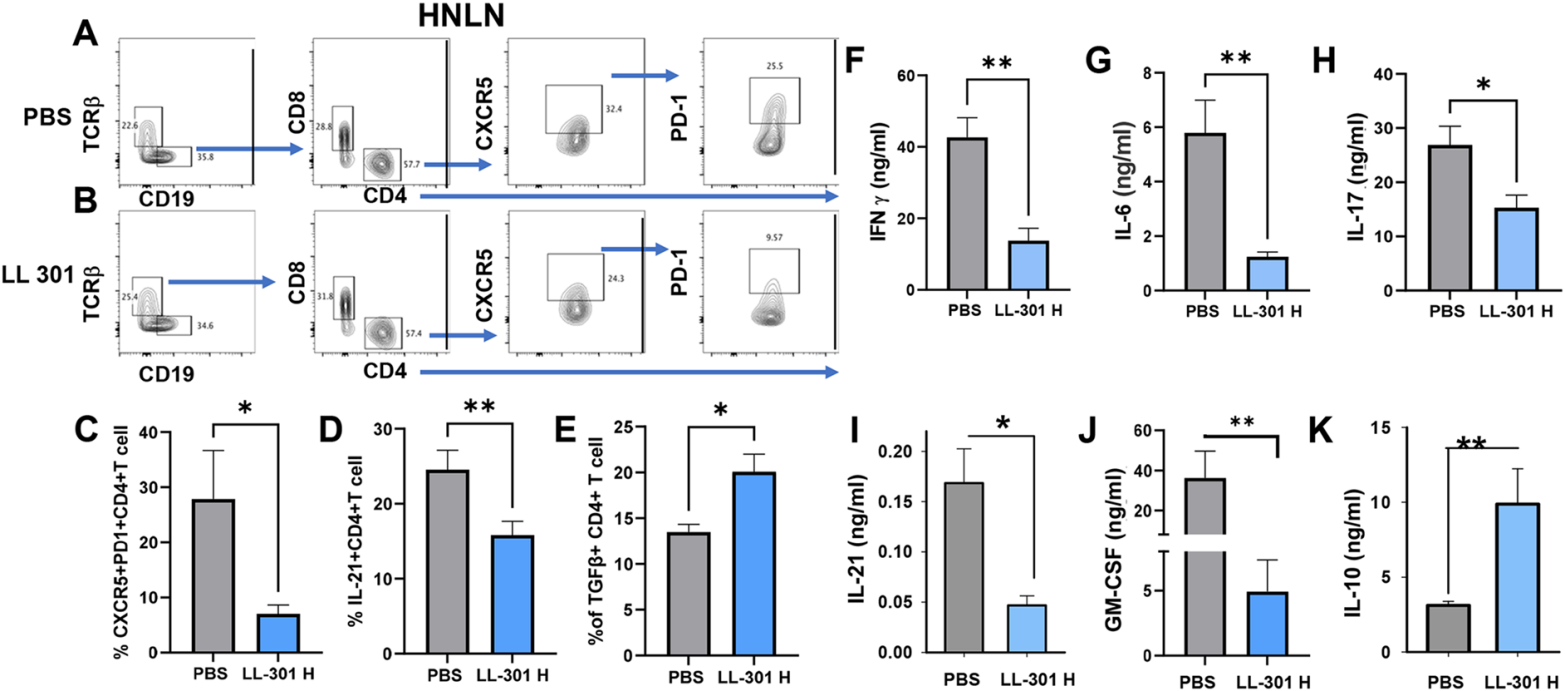
Long-term oral treatment with LL 301 reduces the number of follicular Th (Tfh) cells in the HNLNs and proinflammatory cytokines with concomitant increases in anti-inflammatory cytokines. Groups of 6-week-old SjS (7–8 mice/group) females were orally dosed with 5 × 10^9^ CFUs (high dose) of LL 301 or PBS following a regimen similar to the one described in Fig. 1 with additional doses administered every 3 weeks. The study was terminated at 31 weeks of age (one week after the last dose), and HNLNs were examined levels of (**A-C**) Tfh cells by flow cytometry analysis. Flow cytometry plots representative of HNLN Tfh cells are shown for (**A**) PBS- and (**B**) LL 301-treated mice. The percent (**C**) CXCR5^+^ PD-1^+^ and (**D**) IL-21^+^ CD4^+^ T cells are depicted. (**E**) Flow cytometry analysis was also done for the detection of the percentage of TGF-β^+^ CD4^+^ T cells. (**F–K**) Purified HNLN lymphocytes from PBS- and LL 301-treated females were stimulated with anti-CD3 and anti-CD28 mAbs for 4 days, and culture supernatants were analyzed by cytokine-specific ELISAs. Treatment with LL 301 suppressed production of proinflammatory cytokines (**F**) IFN-γ, (**G**) IL-6, (**H**) IL-17, (**I**) IL-21, and (**J**) GM-CSF. (**K**) LL 301 treatment enhanced IL-10 production. Depicted are the means ± SEM; **P < 0.01 and *P < 0.05 versus PBS-dosed mice.

Tfh cells aid B cells by supporting the formation of germinal centers for maturation of antibody responses, and patients with primary SjS exhibit increased circulating Tfh cells^44^. Given this finding, analyses were conducted to ascertain LL 301’s capacity to inhibit Tfh cells in SjS females. Groups of 6 week-old females were orally dosed with PBS or 5 × 10^9^ CFUs LL 301, and additional doses were administered at 3-week intervals. Upon termination of the study at 31 weeks of age, Tfh cell analyses were performed. HNLN lymphocytes were gated on TCRβ^+^ CD4^+^ T cells and stained for Tfh cells (Fig. 6A, B). Significant reductions in CXCR5^+^ PD-1^+^ CD4^+^ T cells were observed for those SjS females treated with LL 301 relative PBS-treated mice (Fig. 6A–C). Since Tfh cells produce IL-21, analysis of the IL-21^+^ CD4^+^ T cells revealed their percentages were also significantly reduced in the HNLNs from LL 301-treated SjS females (Fig. 6D). The diminished Tfh cell response subsequent LL 301 treatment is consistent with the observed significant increase in TGF-β^+^ CD4^+^ T cells compared to PBS controls (Fig. 6E). HNLN lymphocytes were subsequently stimulated in vitro with anti-CD3 plus anti-CD28 mAbs, and collected culture supernatants from LL 301-treated SjS females showed significantly reduced levels of IFN-γ, IL-6, IL-17, IL-21, and GM-CSF relative to those T cells from PBS controls (Fig. 6F–J). IL-10 levels were significantly increased in for HNLN lymphocytes from LL 301-treated SjS mice (Fig. 6K).

**Figure 7:**
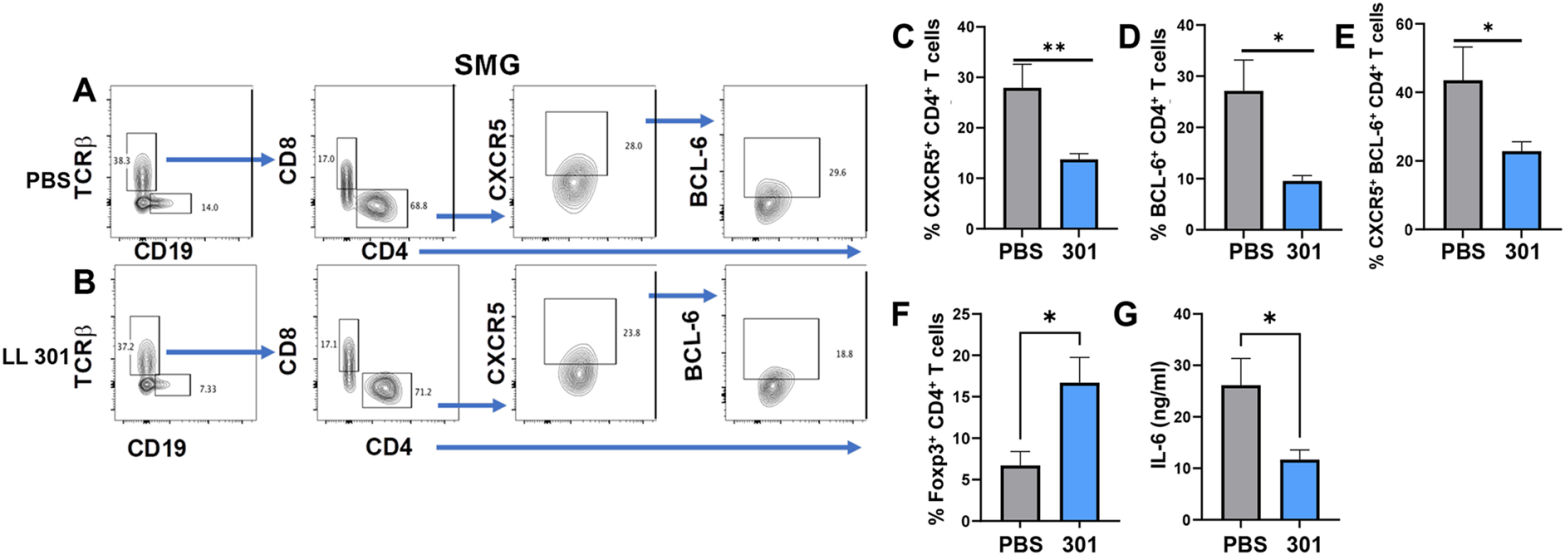
Long-term oral treatment with LL 301 reduces the number of Tfh cells in the SMGs and proinflammatory cytokines. SMGs were isolated from the same mice used in Fig. 6. Upon study termination at 31 weeks of age (one week after the last dose), SMGs were examined levels of (**A–E**) Tfh cells by flow cytometry analysis. Flow cytometry plots representative of SMG Tfh cells are shown for (**A**) PBS- and (**B**) LL 301-treated mice. The percentage of SMG (**C**) CXCR5^+^, (**D**) BCL-6^+^, and (**E**) CXCR5^+^ BCL-6^+^ CD4^+^ T cells are shown. (**F**) Flow cytometry analysis was also done for the detection of the percentage of Foxp3^+^ CD4^+^ T cells. (**G**) Purified SMG lymphocytes from PBS- and LL 301-treated females were stimulated with anti-CD3 and anti-CD28 mAbs for 4 days, and culture supernatants were analyzed for IL-6 production by cytokine-specific ELISA. Depicted are the means ± SEM; **P < 0.01 and *P < 0.05 versus PBS-dosed mice.

SMGs from LL 301- and PBS-treated SjS females were also evaluated for the presence of Tfh cells and Tregs. SMG lymphocytes were gated on TCRβ^+^ CD4^+^ T cells and stained for Tfh cells expressing CXCR5 and BCL-6 (Fig. 7A, B). The percentages of CXCR5^+^ CD4^+^ and BCL6^+^ CD4^+^ T cells were significantly less in the LL 301-treated group compared to PBS-dosed mice (Fig. 7A–D). Likewise, the CXCR5^+^ BCL6^+^ CD4^+^ T cells were also significantly reduced in SMGs from LL 301-treated mice (Fig. 7E). LL 301 treatment positively impacted Treg presence noted by the increased percentage of Foxp3^+^ CD4^+^ T cells in the SMGs relative to those from PBS-treated mice (Fig. 7F). SMG lymphocytes from LL 301-treated SjS females that were restimulated with anti-CD3 plus anti-CD28 mAbs showed a reduction in proinflammatory IL-6 production relative to those lymphocytes from PBS control mice (Fig. 7G).

## Discussion

SjS remains problematic with no current therapies addressing the deficits in regulatory cells to defend against autoimmune attack. The development of oral therapeutics that stimulate Tregs and other regulatory cells to treat autoimmune diseases has the potential to reverse disease. Past studies using CFA/I fimbriae as an oral therapeutic have demonstrated the capabilities of such protein to limit inflammatory T cells in a number of autoimmune disease settings^31–40^. Hence, the development of LL 301 enabling CFA/I protein expression from the *Lactococcus* chromosome averts episomal loss, and represents a cell line that can be tested in humans. The advantage of using *L. lactis* as a delivery vehicle is because of its generally regarded as safe (GRAS) status^41^,and has already been approved for use in foods^45,46^ and treating diseases^45–51^. Another advantage of lactococcal expression is that it avoids the tedious process of isolating and purifying intact CFA/I fimbriae. Moreover, bacterial delivery is more potent and efficient requiring less CFA/I protein to suppress autoimmune disease^36,37^.

The production of CFA/I protein by the LL 301 strain is similar to that of LL-CFA/I. As a result, LL 301 should be as effective as LL-CFA/I, which has previously been shown to protect against collagen-induced arthritis^37^ and SjS^40^. Thus, a comparison study was performed in young SjS females, and showed that LL 301 mitigated salivary flow loss as with LL-CFA/I over the course of 18 weeks. Treatment with the medium dose of LL 301 also lessened the development of ANA responses consistent with previous observations subsequent treatment with LL-CFA/I^40^. LL 301 proved effective in reducing the frequency of proinflammatory IFN-γ^+^ and IL-17^+^ CD4^+^ T cells with concomitant increases in anti-inflammatory IL-10^+^ and TGF-β^+^ CD4^+^ T cells. Reductions in Th1 and Th17 cytokines were primarily observed in the HNLNs, which directly drains the salivary glands. IL-10 production was also reduced in the HNLNs, which may have been attributed to lesser proinflammatory cytokines being induced during the polyclonal stimulation of T cells. Further study was pursued to ascertain the influence of LL 301 upon earlier phase of SjS development until 18 weeks of age. Using 3-week intervals beginning at 5 weeks of age, SjS females showed saliva flow mitigation when treated with either 5 × 10^8^ or 5 × 10^9^ CFUs of LL 301 unlike PBS- and WT LL-treated females noted by their significant reductions in saliva flow. Histological examination of the SMGs further confirmed that LL 301 could reduce the number of foci and foci area of inflammation. Thus, mitigation of the saliva flow was attributed to lessened disease pathology. As with LL-CFA/I^40^, LL 301 showed Treg augmentation, particularly by Foxp3^+^ CD25^+^ CD4^+^ T cells relative to WT LL-treated SjS females. Further inquiry revealed that the CD25^+^ CD4^+^ T cells contain a subset of TGF-β^+^ T cells that also contain anti-inflammatory activity. The capacity to induce or convert Tregs in SjS may be critical. SjS patients have been shown to exhibit deficits in Tregs in their labial salivary glands^52,53^ and peripheral blood^52–54^. Human Tregs from SjS patient were also found to be unresponsive to IL-2 stimulation due to reduced phosphorylation of STAT5^55^, but were able to reduce IL-17^+^ CD4^+^ T cells^56^.

Foxp3 expression varied with times of detection subsequent treatment with LL 301. Our evidence also showed that Tregs did not always express Foxp3, although increases in TGF-β^+^ Foxp3^-^ CD4^+^ T cells were detected. Such evidence of Treg variability has also been previously seen upon *Salmonella*-CFA/I treatment of mice induced with arthritis, whereas both IL-10^+^ Foxp3^+^ CD39^+^ CD4^+^ T cells and TGF-β^+^ Foxp3^-^ CD39^+^ CD4^+^ T cells were co-induced, and were also found to be interconvertible^35^. Adoptive transfer of Foxp3^GFP+^ CD39^+^ CD4^+^ T cells showed a reduction in Foxp3^GFP+^ and IL-10 expression, and vice versa, adoptive transfer of Foxp3^GFP-^ CD39^+^ CD4^+^ T cells showed conversion into IL-10^+^ Foxp3^GFP+^ CD39^+^ CD4^+^ T cells^35^. Importantly, both Treg subsets were needed to adoptively transfer protection. Such interconversion of Tregs demonstrate these cells are dynamic and vary in their expression of Foxp3 as others have also shown^57,58^. Few experimental studies have examined Tregs in SjS animal models. Low-dose IL-2 proved effective in stimulating Foxp3^+^ CD25^+^ CD4^+^ T cells to lessen salivary flow loss in NOD mice^59,60^.

An important finding from the current studies is that LL 301 can intervene with ongoing SjS. Two doses of LL 301 proved sufficient in restoring salivary flow. Although it was less able to impact SMG pathology due to the damage already caused by disease, LL 301 did prove effective in reducing proinflammatory IFN-γ^+^ and IL-17^+^ CD4^+^ T cells despite no significant change in Foxp3^+^ CD25^+^ CD4^+^ T cells. However, these data show that LL 301’s impact is immediate since only two doses were used. This discovery has significant clinical implications since diagnosis of SjS often comes long after the disease is well-established, and having a therapeutic available to interrupt further pathological changes is encouraging. Additional study will be needed to assess whether long-term treatments can prevent further tissue damage.

Tfh cells have also been implicated in contributing to SjS disease exacerbation in their role to expand autoimmune B cells in salivary glands^23,24,61,62^. Normally found associated with B cell follicles, Tfh cells are believed to aid B cells in their expansion and differentiation into plasma cells and memory B cells^63,64^. IL-2 impacts both Tregs and conventional CD4^+^ T cells, but can suppress Tfh cell differentiation^63,64^. In contrast, IL-6 reduces the expression of IL-2Rβ to facilitate Tfh cell expansion^65^. The presented data demonstrates that LL 301 increases Treg presence in lymphoid tissues and in the SMGs. The increased Tregs had a noted effect in reducing the Tfh cells in both the HNLNs and in the SMG. LL 301 also reduced the percentage of IL-21-producing CD4^+^ T cells, a cytokine associated with Tfh cells^63,64^. The production of IL-21 by HNLN T cells was also notably reduced. The reduced Tfh cell presence associated with reduced IL-6 production by both the HNLNs and SMG lymphocytes is indicative that LL 301 is exerting a regulatory effect upon these inflammatory cells. Furthermore, the observed Tfh phenotypes in the SjS mice appeared heterogeneous as others have ascertained^63,64^. In the HNLNs, these were found to be CXCR5^+^ PD-1^+^ CD4^+^ T cells, but their expression of PD-1 by SMG Tfh cells appeared unchanged with or without LL 301 treatment, and primarily were CXCR5^+^ Bcl-6^+^ CD4^+^ T cells. Bcl-6 is a transcription factor, and its activity is essential for Tfh cell differentiation, maintenance, and function^63^. As such, Bcl-6, was markedly expressed by SMG Tfh cells from diseased SjS females, and, LL 301 treatment reduced this population.

In summary, LL 301 proved effective in reducing inflammatory cell infiltration into the SMGs via the stimulation of Tregs and their action by the production of TGF-β and IL-10. Importantly, LL 301 can act therapeutically by reducing inflammatory cell infiltration into the SMGs and restore salivary flow. This study reveals the presence of Tfh cells in diseased C57BL/6.NOD-*Aec1Aec2* mice, and oral LL 301 treatments markedly reduced Tfh cells in both the HNLNs and SMGs. Such findings further demonstrate that orally administered CFA/I protein can intervene to reduce inflammatory diseases. Moreover, our findings support strategies for a robust pharmacodynamic and biomarker assessment plan to speed future clinical development endeavors.

## Methods

### Generation of *Lactococcus lactis* 301 strain and culture conditions

The CFA/I expression cassette was integrated in *L. lactis* genome using standard double homologous recombination methods^66,67^. The *cfa/I* expression operon was synthesized (Blue Heron Biotech, Bothell, WA), and cloned into the pRISE1.2 plasmid (Rise Therapeutics, MD), which contains a temperature-sensitive origin of replication. After transformation into *L. lactis*, cells were initially grown at 30 °C, and then the temperature was shifted to 37 °C to select for integrant cells. The integration occurred at the thymidylate synthase (*thyA*) locus, hence replacing *thyA* from the *L. lactis* chromosome. The purpose of targeting and deleting the *thyA* gene is to allow for containment; that is, the CFA/I expressing *L. lactis* will not propagate without thymidine supplementation^68^. Stability of the integration event was confirmed by growing the selected clones under non-selective conditions in M17 media supplemented with 20 µg/ml thymidine for 100 generations. WT *L. lactis* (LL) was used as a negative control in treating SjS mice. Western blot analysis was performed to confirm expression of CFA/I protein. Purified recombinant CFA/I fimbriae was obtained as previously described^36^, and detected using a rabbit anti-CFA/1 fimbriae antibody (produced in-house). The rabbit anti-CFA/I fimbriae antibody was detected with horseradish peroxidase conjugated goat anti-rabbit IgG (H + L) (Jackson ImmunoResearch, West Grove, PA).

The generation of *L. lactis*-CFA/I was described previously^37^. Briefly, starter small cultures were grown overnight 30 °C, and next day large culture induced with 0.5 µg/mL nisin (Sigma-Aldrich, St. Louis, MO). Four hours after induction, LL-CFA/I was gently washed twice with sterile PBS prior to oral gavage.

### Mice and oral gavage

Using a genetic model for SjS^10^, C57BL/6.NOD-*Aec1Aec* (SjS) female mice were bred and used for these studies. All mice were housed under specific pathogen-free conditions, and provided with food and water ad libitum. Prior to oral administration of lactococcal strains, SjS mice were pretreated orally with sterile 10% sodium bicarbonate solution to neutralize stomach acid. After 10 min, mice proceeded to receive oral gavage with sterile PBS or the indicated doses (5 × 10^7^ to 5 × 10^9^ CFUs) of WT LL, LL 301, or LL-CFA/I. All of the studies involving animals adhered to the recommendations in the Guide for the Care and Use of Laboratory Animals of the National Institutes of Health; were carried out in accordance with relevant guidelines and regulations for ethical and humane treatment of animals; and approved by the University of Florida Institutional Animal Care and Use Committee. The presented study is reported in accordance with ARRIVE guidelines.

### Measurement of salivary flow rate (SFR)

Individual mice were weighed and given an intraperitoneal (IP) injection of 100 μl of a mixture containing isoproterenol (Sigma-Aldrich) (0.2 mg/1 ml of PBS) and pilocarpine hydrochloride (Sigma-Aldrich) (0.05 mg/1 ml in PBS). To measure stimulated flow rates, saliva was collected for 10 min from the oral cavity of each mouse using a micropipette as previously described^40^. To calculate SFRs, the volume of each saliva sample was measured.

### Histology

To assess the degree of inflammation of salivary glands, tissues were fixed in 10% phosphate-buffered formalin (Leica Biosystems, Richmond, IL) for 24 h, and then these were embedded in paraffin and sectioned at 50–100 μm deep at a thickness of 5 μm. Paraffin-embedded sections were deparaffinized by immersing in xylene, followed by dehydration in ethanol, and tissue sections were stained with hematoxylin and eosin (H&E) dye (UF College of Veterinary Medicine Histology Tech Services, Gainesville, FL). To measure the extent of leukocyte infiltration into the salivary glands, a single histological section per gland per mouse was scanned using an Aperio ScanScope (Aperio, San Diego, CA) slide digitizer at 20× magnification as previously described^40^. Sections containing leukocyte-infiltrated regions were identified and calculated using the Aperio ImageScope software. The extent of the infiltrate varied between 1 and in 4 mm^2^, and samples from each mouse for each treatment group were calculated.

### Serum antinuclear antibody (ANA) determinations

To measure the presence of ANA levels, individual serum from mice treated with PBS or LL 301 was examined using the HEp-2 ANA kit (Inova Diagnostics, Inc., San Diego, CA, USA) by following manufacturer’s instructions^14–16,69^. Serum samples, each diluted 1:40, were incubated on HEp-2-fixed substrate slides for one hr at room temperature in a humidified chamber. After three 5-min washes with PBS, the slides were treated with a 1:100 dilution of Alexa 488 goat anti-mouse IgG (H + L) (Life Technologies) for 45 min at room temperature. After three washes, Vectashield DAPI mounting medium (Vector Laboratories, Burlingame, CA, USA) was applied, and overlaid with a glass coverslip. Fluorescence was detected by fluorescence Nikon microscopy at 400× magnification, and all images were obtained with exposure of 200 ms.

### Lymphocyte cell culture

Head and neck lymph node (HNLNs), mesenteric LNs (MLNs), and spleens were aseptically removed, and single cell suspensions were prepared as previously described^70^. Briefly, tissues were homogenized using a single sterile, stainless steel bead (McMaster-Carr, Elmhurst, IL) in sterile 2.0 ml locking microfuge tube (Qiagen) shaken in a Tissue Lyser (QIAGEN), then filtered through 80 μm nylon mesh (Component Supply Company, Sparta TN), and washed for 5 min at 4 °C in incomplete media (ICM): RPMI 1640 with L-glutamine (Genesee Scientific, El Cajon, CA); 10 mM HEPES buffer (Caisson Labs); and 10 mM penicillin/streptomycin (Caisson Labs). Splenic red blood cells were lysed using 5 ml of ammonium-chloride-potassium (ACK) buffer (0.15 M NH_4_Cl, 10 mM KHCO_3_, 0.1 mM Na_2_EDTA) for 5 min. Lymphocytes were cultured in a complete medium (CM): ICM plus 10% fetal bovine serum (Atlanta Biologicals, Oakwood, Georgia) and supplemented with 1 mM sodium pyruvate, and 0.1 mM nonessential amino acids (Invitrogen, Carlsbad, CA. For restimulation assays, lymphocytes were cultured in triplicate using 10^6^ cells/well for 2–4 days at 37 °C in 96-well, round-bottomed tissue culture plates (Millipore, Billerica, MA) coated with 5 µg/ml anti-CD3 mAb (clone 17A2; Invitrogen, Carlsbad, CA, USA) plus 2.5 µg/ml of soluble anti-CD28 mAb (clone 37.51; Invitrogen). Cell culture supernatants were collected and then stored at −20 °C until assayed by cytokine-specific ELISAs.

### Flow cytometry

For flow cytometry analysis, restimulated lymphocytes were treated with 5 µg/mL brefeldin A (Biovision, San Francisco, CA, USA) for 3–4 h to block cytokine release. Splenic and LN lymphocytes were subjected to a viability stain using a LIVE/DEAD Fixable Blue Dead Cell Stain Kit, for UV excitation (ThermoFisher). Cells were then washed with Dulbecco’s PBS (Gibco, ThermoFisher**)** plus 10% fetal bovine serum (Atlanta Biologicals**),** and labeled with mAbs specific for TCR-β, CD4, CD8α, CD19, CD25, CXCR5 (clone L138D7), PD-1 (clone 29F.1A12), TGF-β (BioLegend, San Diego, CA), and CD39 (eBioscience, San Diego, CA). The mAb clones were the same as those previously described^39^ unless indicated. Cells were then fixed and permeabilized using the True-Nuclear Transcription Factor Buffer Set (BioLegend) and labeled with mAbs specific for IFN-γ (clone XMG1.2), IL-10 (clone JES5-16F3), IL-17 (clone TC11-18H10.1), Bcl-6 (clone 7D1; BioLegend); IL-6 (clone MP5-20F3; BD Pharmingen, San Jose, CA); IL-21 (clone mhalx21), GM-CSF (clone MP1-22E9), and Foxp3 (clone FJK-16a; eBioscience). Fluorescence was acquired on a Fortessa flow cytometer (Becton Dickinson Franklin Lakes, NJ), using FACSDiva software (Becton Dickinson). All samples were analyzed using FlowJo software (BD Biosciences, Ashland, OR).

### Cytokine ELISA

At termination of the studies, harvested HNLN, MLN, and splenic lymphocytes were restimulated and cultured as described above. Cytokine capture ELISAs were used to quantify levels of IFN-γ, GM-CSF, IL-6, IL-10, IL-17, and TGF-β present in culture supernatants. Identical mAb pairs and methods were used as previously described^40^. For IL-21, a goat anti-mouse IL-21 and biotinylated goat anti-mouse IL-21 (R&D Systems) were used. A horseradish peroxidase conjugated goat anti-biotin Ab (Vector Laboratories) was used for the tertiary Ab. The color reaction was developed using and ABTS peroxidase substrate (Moss, Inc., Pasadena, ME), and absorbances were measured using an Epoch Microplate Spectrophotometer (BioTek Instruments, Winooski, VT). Cytokine concentrations were extrapolated from standard curves generated by recombinant murine cytokines IFN-γ and IL-21 (Peprotech, Cranbury, NJ), IL-6 (BD Pharmingen), IL-10, IL-17, GM-CSF (eBioscience, San Diego, CA), and TGF-β (R&D Systems, Minneapolis, MN, USA).

### Statistics

A power analysis was conducted, and found that 5 mice per group were needed to show a significant difference for at least a 20% change in SFR. All presented data are the mean ± standard error of the mean (SEM). Statistical significance was tested using GraphPad Prism 8 (Prism, Irvine, CA). One-way ANOVA with Tukey’s multiple comparisons test were used to compare FACS data, cell counts, cytokine production, and salivary flow rates. All results are discerned to the 95% confidence interval.

### Supplementary Information

Supplementary Figure 1.

## Data Availability

The original contributions presented in the study are included in the article. Further inquiries can be directed to the corresponding author.

## References

[1] Pontarini E, Lucchesi D, Bombardieri M (2018). Current views on the pathogenesis of Sjögren's syndrome. Curr. Opin. Rheumatol..

[2] Psianou K (2018). Clinical and immunological parameters of Sjögren's syndrome. Autoimmun. Rev..

[3] Nocturne G, Mariette X (2013). Advances in understanding the pathogenesis of primary Sjögren's syndrome. Nat. Rev. Rheumatol..

[4] Moerman RV, Bootsma H, Kroese FG, Vissink A (2013). Sjögren's syndrome in older patients: A etiology, diagnosis and management. Drugs Aging.

[5] Kassan SS, Moutsopoulos HM (2004). Clinical manifestations and early diagnosis of Sjögren’s syndrome. Arch. Intern. Med..

[6] Helmick, C.G., Felson, D.T., Lawrence, R.C., Gabriel, S., Hirsch, R., Kwoh, C.K., Liang, M.H., Kremers, H.M., Mayes, M.D., Merkel, PA., Pillemer, S.R., Reveille, J.D., Stone, J.H., & National Arthritis Data Workgroup. Estimates of the prevalence of arthritis and other rheumatic conditions in the United States. Part I. *Arthritis Rheum*. **58**, 15–25 (2008). 10.1002/art.23177 18163481

[7] Nguyen CQ, Peck AB (2009). Unraveling the pathophysiology of Sjögren syndrome-associated dry eye disease. Ocul. Surf..

[8] Delaleu N, Nguyen CQ, Peck AB, Jonsson R (2011). Sjögren's syndrome: Studying the disease in mice. Arthritis Res. Ther..

[9] Ridgway, W. M. *et al*. Chapter 6 Gene–gene interactions in the NOD mouse model of type 1 diabetes. *Adv. Immunol.* **100**, 151–75 (2008). 10.1016/S0065-2776(08)00806-7 19111166

[10] Cha S, Nagashima H, Brown VB, Peck AB, Humphreys-Beher MG (2002). Two NOD Idd-associated intervals contribute synergistically to the development of autoimmune exocrinopathy (Sjögren's syndrome) on a healthy murine background. Arthritis Rheum..

[11] Nguyen CQ, Hu MH, Li Y, Stewart C, Peck AB (2008). Salivary gland tissue expression of interleukin-23 and interleukin-17 in Sjögren’s syndrome: Findings in humans and mice. Arthritis Rheum..

[12] Mavragani CP, Nezos A, Moutsopoulos HM (2013). New advances in the classification, pathogenesis and treatment of Sjogren's syndrome. Curr. Opin. Rheumatol..

[13] Ramos-Casals M, Brito-Zerón P, Sisó-Almirall A, Bosch X, Tzioufas AG (2012). Topical and systemic medications for the treatment of primary Sjögren's syndrome. Nat. Rev. Rheumatol..

[14] Cornec D, Devauchelle-Pensec V, Tobón GJ, Pers JO, Jousse-Joulin S, Saraux A (2012). B cells in Sjögren's syndrome: From pathophysiology to diagnosis and treatment. J. Autoimmun..

[15] Meiners PM, Vissink A, Kallenberg CG, Kroese FG, Bootsma H (2011). Treatment of primary Sjögren's syndrome with anti-CD20 therapy (rituximab). A feasible approach or just a starting point?. Expert Opin. Biol. Ther..

[16] Perosa F, Prete M, Racanelli V, Dammacco F (2010). CD20-depleting therapy in autoimmune diseases: From basic research to the clinic. J. Intern. Med..

[17] Fox RI, Fox CM, Gottenberg JE, Dörner T (2021). Treatment of Sjögren's syndrome: Current therapy and future directions. Rheumatology (Oxford).

[18] Grigoriadou S, Chowdhury F, Pontarini E, Tappuni A, Bowman SJ, Bombardieri M (2019). B cell depletion with rituximab in the treatment of primary Sjögren's syndrome: What have we learnt?. Clin. Exp. Rheumatol..

[19] Bowman SJ, Everett CC, O'Dwyer JL, Emery P, Pitzalis C, Ng WF, Pease CT, Price EJ, Sutcliffe N, Gendi NST, Hall FC, Ruddock SP, Fernandez C, Reynolds C, Hulme CT, Davies KA, Edwards CJ, Lanyon PC, Moots RJ, Roussou E, Giles IP, Sharples LD, Bombardieri M (2017). Randomized controlled trial of rituximab and cost-effectiveness analysis in treating fatigue and oral dryness in primary Sjogren's syndrome. Arthritis Rheumatol..

[20] Devauchelle-Pensec V, Mariette X, Jousse-Joulin S, Berthelot JM, Perdriger A, Puéchal X, Le Guern V, Sibilia J, Gottenberg JE, Chiche L, Hachulla E, Hatron PY, Goeb V, Hayem G, Morel J, Zarnitsky C, Dubost JJ, Pers JO, Nowak E, Saraux A (2014). Treatment of primary Sjögren syndrome with rituximab: A randomized trial. Ann. Intern. Med..

[21] Tsuboi, H., Matsumoto, I., Hagiwara, S., Hirota, T., Takahashi, H., Ebe, H., Yokosawa, M., Yagishita, M., Takahashi, H., Kurata, I., Ohyama, A., Honda, F., Asashima, H., Miki, H., Umeda, N., Kondo, Y., Hirata, S., Saito, K., Tanaka, Y., Horai, Y., Nakamura, H., Kawakami, A. & Sumida, T. Effectiveness of abatacept for patients with Sjögren's syndrome associated with rheumatoid arthritis. An open label, multicenter, one-year, prospective study: ROSE (Rheumatoid Arthritis with Orencia Trial toward Sjögren's syndrome Endocrinopathy) trial. *Mod. Rheumatol*. **26**, 891–899 (2016). 10.3109/14397595.2016.1158773 27459020

[22] Adler S, Körner M, Förger F, Huscher D, Caversaccio MD, Villiger PM (2013). Evaluation of histologic, serologic, and clinical changes in response to abatacept treatment of primary Sjögren’s syndrome: A pilot study. Arthritis Care Res. (Hoboken).

[23] Chen W, Yang F, Xu G, Ma J, Lin J (2021). Follicular helper T cells and follicular regulatory T cells in the immunopathology of primary Sjögren's syndrome. J. Leukoc. Biol..

[24] Verstappen GM, Meiners PM, Corneth OBJ, Visser A, Arends S, Abdulahad WH, Hendriks RW, Vissink A, Kroese FGM, Bootsma H (2017). Attenuation of follicular helper T cell-dependent B cell hyperactivity by abatacept treatment in primary Sjogren's syndrome. Arthritis Rheumatol..

[25] Tsuboi H, Toko H, Honda F, Abe S, Takahashi H, Yagishita M, Hagiwara S, Ohyama A, Kondo Y, Nakano K, Tanaka Y, Shimizu T, Nakamura H, Kawakami A, Fujieda Y, Atsumi T, Suzuki Y, Kawano M, Nishina N, Kaneko Y, Takeuchi T, Kobayashi H, Takei M, Ogasawara M, Tamura N, Takasaki Y, Yokota K, Akiyama Y, Mimura T, Murakami K, Mimori T, Ohshima S, Azuma N, Sano H, Nishiyama S, Matsumoto I, Sumida T (2022). Abatacept ameliorates both glandular and extraglandular involvements in patients with Sjögren's syndrome associated with rheumatoid arthritis: Findings from an open-label, multicenter, 1-year, prospective study: The ROSE (Rheumatoid Arthritis with Orencia Trial Toward Sjögren's Syndrome Endocrinopathy) and ROSE II trials. Mod. Rheumatol..

[26] de Wolff L, van Nimwegen JF, Mossel E, van Zuiden GS, Stel AJ, Majoor KI, Olie L, Los LI, Vissink A, Spijkervet FKL, Verstappen GMPJ, Kroese FGM, Arends S, Bootsma H (2022). Long-term abatacept treatment for 48 weeks in patients with primary Sjögren's syndrome: The open-label extension phase of the ASAP-III trial. Semin. Arthritis Rheum..

[27] Edelman R (1993). Immunization of rabbits with enterotoxigenic *E. coli* colonization factor antigen (CFA/I) encapsulated in biodegradable microspheres of poly (lactide-*co*-glycolide). Vaccine.

[28] Evans DG, Graham DY, Evans DJ, Opekun A (1984). Administration of purified colonization factor antigens (CFA/I, CFA/II) of enterotoxigenic *Escherichia coli* to volunteers. Gastroenterology.

[29] Schmidt M, Kelly EP, Tseng LY, Boedeker EC (1985). Towards an oral *E. coli* pilus vaccine for traveler’s diarrhea: Susceptibility to proteolytic digestion. Gastroenterology.

[30] Reid RH, Boedeker EC, McQueen CE, Davis D, Tseng L-Y, Kodak J, Sau K, Wilhelmesen CL, Nellore R, Dalal P, Bhagat HR (1993). Preclinical evaluation of microencapsulated CFA/II oral vaccine against enterotoxigenic *E. coli*. Vaccine.

[31] Jun S, Gilmore W, Callis G, Rynda A, Haddad A, Pascual DW (2005). A live diarrheal vaccine imprints a Th2 cell bias and acts as an anti-inflammatory vaccine. J. Immunol..

[32] Ochoa-Repáraz J, Riccardi C, Rynda A, Jun S, Callis G, Pascual DW (2007). Regulatory T cell vaccination without autoantigen protects against experimental autoimmune encephalomyelitis. J. Immunol..

[33] Ochoa-Repáraz J, Rynda A, Ascón MA, Yang X, Kochetkova I, Riccardi C, Callis G, Trunkle T, Pascual DW (2008). IL-13 production by regulatory T cells protects against experimental autoimmune encephalomyelitis independently of autoantigen. J. Immunol..

[34] Kochetkova I, Trunkle T, Callis G, Pascual DW (2008). Vaccination without auto-antigen protects against collagen II-induced arthritis via immune deviation and regulatory T cells. J. Immunol..

[35] Kochetkova I, Crist K, Callis G, Pascual DW (2011). Segregated regulatory CD39^+^ CD4^+^ T cell function: TGF-β-producing Foxp3^-^ and IL-10-producing Foxp3^+^ cells are interdependent for protection against collagen-induced arthritis. J. Immunol..

[36] Kochetkova I, Thornburg T, Callis G, Holderness K, Maddaloni M, Pascual DW (2014). Oral *Escherichia coli* colonization factor antigen I (CFA/I) fimbriae ameliorate arthritis via IL-35, not IL-27. J. Immunol..

[37] Maddaloni M, Kochetkova I, Jun S, Callis G, Thornburg T, Pascual DW (2015). Milk-based nutraceutical for treating autoimmune arthritis via the stimulation of IL-10- and TGF-ß-producing CD39^+^ regulatory T cells. PLoS One.

[38] Nelson AS, Maddaloni M, Abbott J, Hoffman C, Akgul A, Ohland C, Gharalbeh R, Jobin C, Brusko TM, Pascual DW (2020). Oral therapy with colonization factor antigen I prevents development of type 1 diabetes in non-obese diabetic mice. Sci. Rep..

[39] Nelson AS, Akgul A, Maddaloni M, Bhagyaraj E, Hoffman C, Pascual DW (2021). Oral probiotic promotes indoleamine 2,3-dioxygenase- and TGF-β-producing plasmacytoid dendritic cells to initiate protection against type 1 diabetes. Immunol. Lett..

[40] Akgul A, Maddaloni M, Jun S-M, Nelson AS, Aguilera Odreman V, Hoffman C, Bhagyaraj E, Voigt A, Abott JR, Nguyen CQ, Pascual DW (2021). Stimulation of regulatory T cells with *Lactococcus lactis* expressing enterotoxigenic *E. coli* colonization factor antigen 1 retains salivary flow in a genetic model of Sjögren’s syndrome. Arthritis Res. Ther..

[41] Wells J (2011). Mucosal vaccination and therapy with genetically modified lactic acid bacteria. Annu. Rev. Food Sci. Technol..

[42] Klijn N, Weerkamp AH, de Vos WM (1995). Genetic marking of *Lactococcus lactis* shows its survival in the human gastrointestinal tract. Appl. Environ. Microbiol..

[43] Daniel C, Poiret S, Dennin V, Boutillier D, Pot B (2013). Bioluminescent *Lactobacillus plantarum* and *Lactococcus lactis* to study spatial and temporal persistence in living mice. Appl. Environ. Microbiol..

[44] Szabó K, Jámbor I, Szántó A, Horváth IF, Tarr T, Nakken B, Szodoray P, Papp G (2021). The imbalance of circulating follicular T helper cell subsets in primary Sjögren's syndrome associates with serological alterations and abnormal B-cell distribution. Front. Immunol..

[45] Preisser TM, da Cunha VP, Santana MP, Pereira VB, Cara DC, Souza BM, Miyoshi A (2021). Recombinant *Lactococcus lactis* carrying IL-4 and IL-10 coding vectors protects against type 1 diabetes in NOD mice and attenuates insulitis in the STZ-induced model. J. Diabetes Res..

[46] Tavares LM, de Jesus LCL, da Silva TF, Barroso FAL, Batista VL, Coelho-Rocha ND, Azevedo V, Drumond MM, Mancha-Agresti P (2020). Novel strategies for efficient production and delivery of live biotherapeutics and biotechnological uses of *Lactococcus lactis*: The lactic acid bacterium model. Front. Bioeng. Biotechnol..

[47] Cook DP, Gysemans C, Mathieu C (2018). *Lactococcus lactis* as a versatile vehicle for tolerogenic immunotherapy. Front. Immunol..

[48] Kimoto-Nira H, Nagakura Y, Kodama C, Shimizu T, Okuta M, Sasaki K, Koikawa N, Sakuraba K, Suzuki C, Suzuki Y (2014). Effects of ingesting milk fermented by *Lactococcus* lactis H61 on skin health in young women: A randomized double-blind study. J. Dairy Sci..

[49] Fleming JO, Isaak A, Lee JE, Luzzio CC, Carrithers MD, Cook TD, Field AS, Boland J, Fabry Z (2011). Probiotic helminth administration in relapsing-remitting multiple sclerosis: A phase 1 study. Mult. Scler..

[50] Rottiers P, De Smedt T, Steidler L (2009). Modulation of gut-associated lymphoid tissue functions with genetically modified *Lactococcus lactis*. Int. Rev. Immunol..

[51] Steidler L, Neirynck S, Huyghebaert N, Snoeck V, Vermeire A, Goddeeris B, Cox E, Remon JP, Remaut E (2003). Biological containment of genetically modified *Lactococcus lactis* for intestinal delivery of human interleukin 10. Nat. Biotechnol..

[52] Celenligil H, Kansu E, Ruacan S, Eratalay K, Irkec M (1990). Characterization of peripheral blood and salivary gland lymphocytes in Sjogren's syndrome. Oral Surg. Oral Med. Oral Pathol..

[53] Li X, Qian L, Wang G, Zhang H, Wang X, Chen K, Zhai Z, Li Q, Wang Y, Harris DC (2007). T regulatory cells are markedly diminished in diseased salivary glands of patients with primary Sjogren's syndrome. J. Rheumatol..

[54] Liu MF, Lin LH, Weng CT, Weng MY (2008). Decreased CD4^+^CD25^+bright^ T cells in peripheral blood of patients with primary Sjögren’s syndrome. Lupus.

[55] Keindl M, Davies R, Bergum B, Brun JG, Hammenfors D, Jonsson R, Lyssenko V, Appel S (2022). Impaired activation of STAT5 upon IL-2 stimulation in Tregs and elevated sIL-2R in Sjögren's syndrome. Arthritis Res. Ther..

[56] Luo J, Ming B, Zhang C, Deng X, Li P, Wei Z, Xia Y, Jiang K, Ye H, Ma W, Liu Z, Li H, Yang XP, Dong L (2018). IL-2 inhibition of Th17 generation rather than induction of Treg cells is impaired in primary Sjögren's syndrome patients. Front. Immunol..

[57] Raugh A, Allard D, Bettini M (2022). Nature vs. nurture: FOXP3, genetics, and tissue environment shape Treg function. Front. Immunol..

[58] Li MO, Rudensky AY (2016). T cell receptor signalling in the control of regulatory T cell differentiation and function. Nat. Rev. Immunol..

[59] Wang Y, Feng R, Cheng G, Huang B, Tian J, Gan Y, Jin Y, Miao M, Zhang X, Sun X, He J, Li Z (2022). Low dose interleukin-2 ameliorates Sjögren's syndrome in a murine model. Front. Med. (Lausanne).

[60] Wen J, Zhu F, Yu X, Xie H, Li C (2022). Low-dose interleukin-2 can improve salivary secretion but not lymphocyte infiltration of salivary glands in a murine model of Sjögren's syndrome. BMC Immunol..

[61] Pontarini E, Coleby R, Bombardieri M (2021). Cellular and molecular diversity in Sjogren's syndrome salivary glands: Towards a better definition of disease subsets. Semin. Immunol..

[62] Ren HM, Lukacher AE, Rahman ZSM, Olsen NJ (2021). New developments implicating IL-21 in autoimmune disease. J. Autoimmun..

[63] Walker LSK (2022). The link between circulating follicular helper T cells and autoimmunity. Nat. Rev. Immunol..

[64] Tangye SG, Ma CS (2021). Molecular regulation and dysregulation of T follicular helper cells—Learning from inborn errors of immunity. Curr. Opin. Immunol..

[65] Papillion, A. *et al*. Inhibition of IL-2 responsiveness by IL-6 is required for the generation of GC-TFH cells. *Sci. Immunol.* **4**, eaaw7636 (2019). 10.1126/sciimmunol.aaw7636 PMC6820141 31519812

[66] Parker J, Pollard JW, Friesen JD, Stanners CP (1978). Stuttering: High-level mistranslation in animal and bacterial cells. Proc. Natl. Acad. Sci. U S A.

[67] Leenhouts KJ, Kok J, Venema G (1991). Replacement recombination in *Lactococcus lactis*. J. Bacteriol..

[68] Steidler L (2003). Gene exchange of thyA for interleukin-10 secures live GMO bacterial therapeutics. Discov. Med..

[69] Lee BH, Carcamo WC, Chiorini JA, Peck AB, Nguyen CQ (2012). Gene therapy using IL-27 ameliorates Sjögren's syndrome-like autoimmune exocrinopathy. Arthritis Res. Ther..

[70] Goodwin ZI, Yang X, Hoffman C, Pascual DW (2022). Live mucosal vaccination stimulates potent protection *via* varied CD4^+^ and CD8^+^ T cell subsets against wild-type *Brucella melitensis* 16M challenge. Front. Immunol..

