# Supplementary material for: Treatment with a Lactococcus lactis that chromosomally express E. coli cfaI mitigates salivary flow loss in a Sjögren’s syndrome-like disease
Source: Sci Rep. 2023 Nov 9;13:19489. doi: 10.1038/s41598-023-46557-3 (PMC10636062; doi:10.1038/s41598-023-46557-3)
Supplement: Supplementary file 1 — Supplementary Figure 1. [file 41598_2023_46557_MOESM1_ESM.pdf]

Supplementary Figure 1

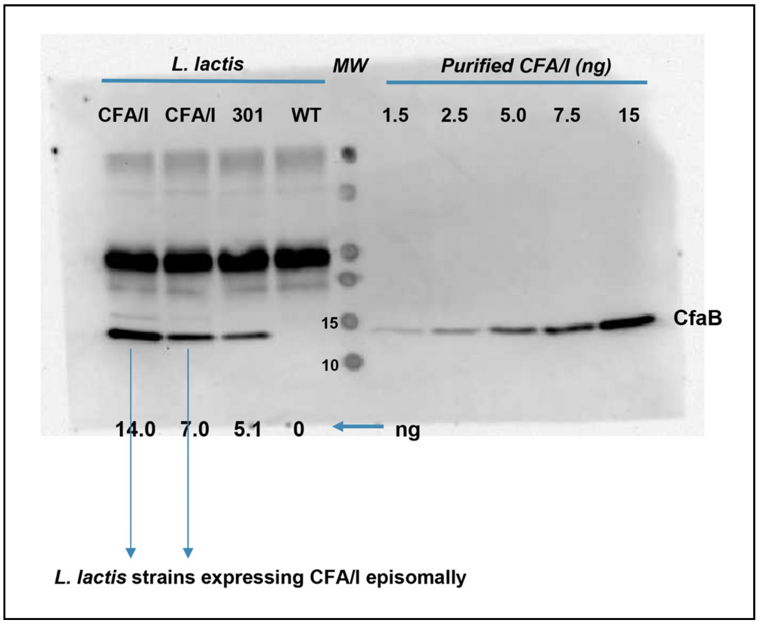

The complete image with edges for the Western blot presented in Figure 1A for *Lactococcus lactis* (LL) 301. A Western blot was performed on whole bacterial extracts from (Lane 1) LL-CFA/I episomal strain generated by Rise Therapeutics; (Lane 2) LL-CFA/I (reference 37); (Lane 3) LL 301; and (Lane 4) wild-type (WT) LL electrophoresed in SDS polyacrylamide gel. The CfaB subunit was detected with a rabbit IgG anti-CFA/I fimbriae (produced in-house), and bands migrated with similar molecular weight (MW) in kilodaltons as purified CFA/I fimbriae. The amount of CfaB produced by 10^9 bacteria is estimated numbers presented at the bottom of the Western blot) from densiometric scan of purified CFA/I fimbriae (right hand side of the image). The larger MW products detected with the rabbit IgG Ab are believed to be other LL proteins, e.g., membrane proteins, since these are equally present in the four strains. Note: Lane 1 is not presented in Figure 1a. In Figure 1a, Lane 1 begins with Lane 2 of the original blot as not to confuse the reader for having two different episomal constructs for LL-CFA/I.
